# Factors associated with first-line antiretroviral treatment failure in adult HIV-positive patients: a case-control study from Ethiopia

**DOI:** 10.1186/s12879-019-4170-5

**Published:** 2019-06-18

**Authors:** Yihienew Mequanint Bezabih, Fekadu Beyene, Woldesellassie M. Bezabhe

**Affiliations:** 1 College of Health Sciences, Arsi University, Arsi, Ethiopia; 2 ONIRIS: The Nantes-Atlantic National College of Veterinary Medicine, Nantes, France; 3 Division of Pharmacy, School of Medicine, University of Tasmania, Tasmania, Australia

**Keywords:** Virologic failure, Antiretroviral, Case-control, HIV, AIDS, Ethiopia

## Abstract

**Background:**

Treatment failure has become a significant challenge in patients taking antiretroviral therapy (ART). The aim of the present study was to identify risk factors for first-line ART failure among patients attending clinical follow-up.

**Methods:**

A 1:2 matched case-control study (by age, sex, and treatment duration since initiated on ART) was conducted from June 2015 to July 2017 on adult patients (aged ≥15 years) who were on ART for at least 6 months. Cases were selected from patients who were switched to second-line ART after first-line ART failure (viral load ≥1000 copies/mL). Controls were randomly selected from patients on first-line ART with viral load < 50 copies/mL. Data were collected using an interview questionnaire, reviewing chart and electronic health records and laboratory tests. Multivariate logistic regression analysis was performed to identify risk factors for treatment failure.

**Results:**

Of the 273 patients who participated in this study, 55% were males. Ninety-one cases were compared with 182 controls. The median age of participants was 40 years and the median duration of treatment since initiated on ART was 69 months. Independent risk factors associated with first-line antiretroviral treatment failure were discontinuation of ART (adjusted odds ratio (AOR) = 9.8, 95% confidence interval (CI): 4.0–23.8), baseline CD4 lymphocyte count ≤50 cells/mm^3^ (AOR = 3.8, 95% CI: 1.5–9.6) and persistent diarrhea (AOR = 4.4, 95% CI: 1.5–13.2). The risk of ART failure was high and comparable whether the duration of ART discontinuation was greater or less than 1 month (crude odds ratio (COR) = 6.3 and 8. 5 respectively, *p*-value < 0.001). Frequent eating of a diet containing wheat or barley (AOR = 2.3, 95% CI: 0.9–5.4) showed a trend to be a risk factor for first-line ART failure (p-value = 0.064).

**Conclusions:**

Our findings underscore the importance of avoiding ART discontinuation of any duration, early initiation of ART and diarrhea management to prevent first-line ART failure.

## Background

Worldwide Human Immunodeficiency Virus (HIV)/Acquired Immune Deficiency Syndrome (AIDS) is a major public health issue. In 2017, over 36.9 million people were living with HIV/AIDS. The most affected continent, Africa, had 25.7 million (70%) people with HIV/AIDS. In Ethiopia, about 710, 000 people were living with the disease as of 2017 [1]. The global antiretroviral therapy (ART) coverage increased progressively from 7% in 2005 to 59% in 2017 [2, 3]. The estimated coverages in 2017 were 60 and 59% for Africa and Ethiopia, respectively [1].

Because of this extensive ART scale-up, treatment failure has become an emerging problem [4, 5]. The prevalence of first-line ART failure differs significantly across countries depending on the criteria (clinical, immunological or virologic) used for its diagnosis. The viral load cut-off points to diagnose treatment failure also vary in different countries. The first-line ART virologic failure rate in Africa was 7.1 (95% CI: 5.1–9.1) per 100 patient-years of follow-up [6]. In Ethiopia, studies reported virologic failure rates ranging from 5.3 to 19% [7–9], and the percentage of patients switched to second-line ART was 1.5% in 2013 [10]. Switching to second-line ART means that patients are receiving more toxic medications. As a result, patients become less adherent, and AIDS progression will be more rapid, both of which make patient monitoring expensive [11–14].

Previous studies identified that suboptimal adherence, adverse drug reactions [15, 16], higher pre-treatment (baseline) HIV viral load level, baseline World Health Organization (WHO) AIDS stage 3 or 4, CD4 lymphocyte count < 50 cells/mm^3^, low body mass index (BMI), drug interactions and dyspepsia as an important risk factors for treatment failure [17–22]. A study with a clear distinction between participants with and without ART failure remains important to identify predictors of treatment failure. To our knowledge, most studies from Ethiopia used clinical and immunologic criteria to assess factors that influence treatment failure. As immunologic and clinical criteria have low sensitivity and specificity to identify true ART failure [23], it could be difficult to identify risk factors based on these two criteria alone. Therefore, this study aimed to investigate risk factors for first-line antiretroviral failure using the virologic (plasma viral load) criteria.

## Methods

### Study setting

This study was carried out at the ART follow-up clinic of Asella Hospital, Ethiopia. The hospital gives clinical service to a total population of about 3.5 million people. There were 3238 patients with HIV/AIDS (including those from the surrounding rural areas and nearby towns) who had an ART follow-up in the hospital of which 102 (3.2%) patients were on second-line ART.

### Study design and participants

**Fig. 1:**
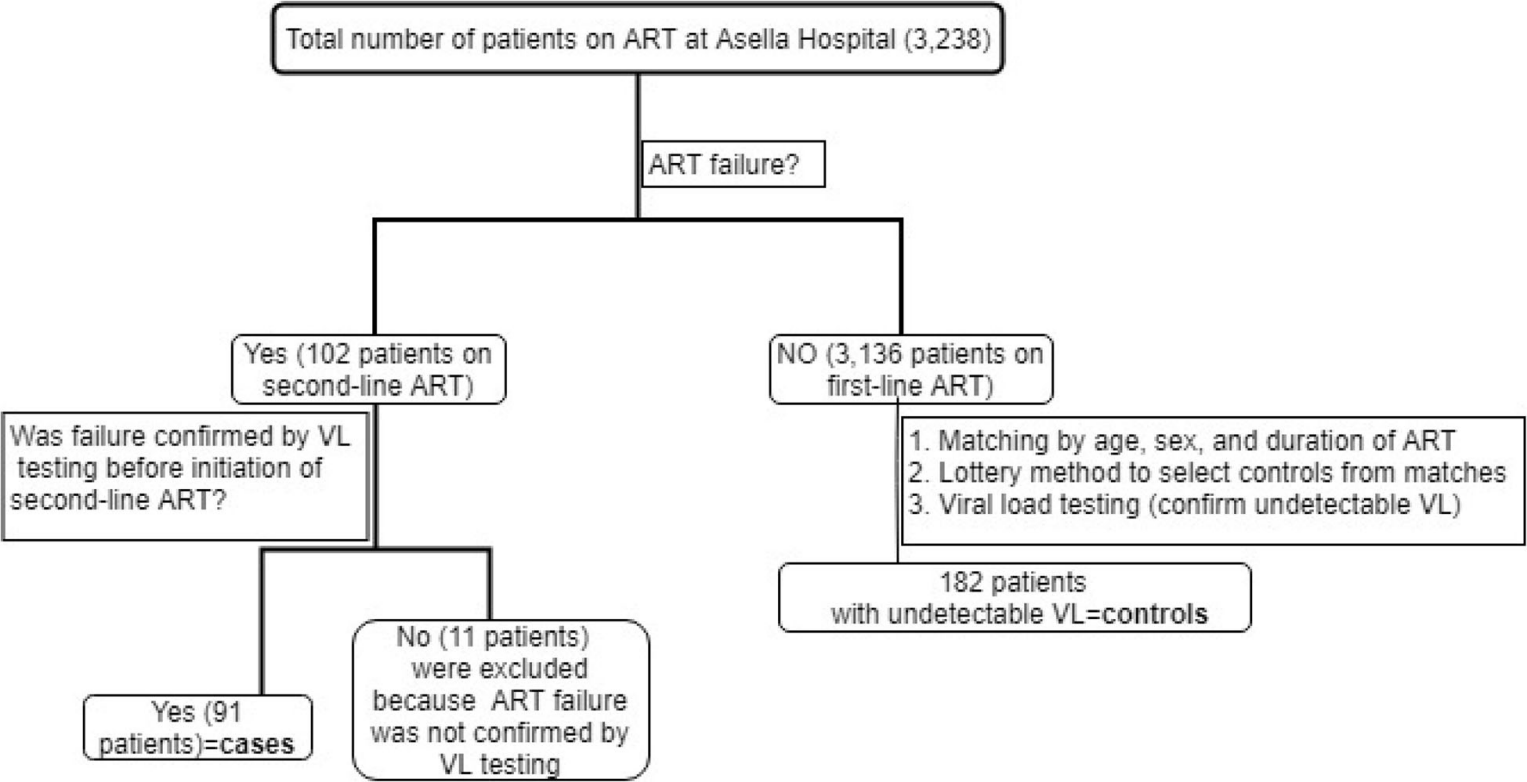
The selection process of study participants. (ART = Antiretroviral therapy, VL = viral load)

A 1:2 matched case-control study (by age, sex and treatment duration since initiated on ART) was conducted between June 2015 to July 2017 (study period). All adult HIV-positive patients (aged ≥15 years) who were on ART for at least 6 months and whose baseline CD4 lymphocyte count and WHO HIV stage details available using the ART electronic health record of Asella Hospital were our target population. This data ranges from January 2005 to May 2017, and only patients not died and had an active follow-up at the time of the study were included. Ninety-one patients who were receiving second-line ART were selected as cases. Treatment failure status for cases was confirmed by two viral load tests (viral load ≥1000 copies/mL), performed 3 months apart as per national guideline, before a change of ART regimen. Controls were 182 patients with undetectable viral load (less than 50 copies/mL) who were taking first-line ART for at least 6 months. Details of the selection process are described below (Fig. 1).

For each case, two controls were selected using a lottery method after being matched by age, sex, and duration on ART. We used the following technique for selecting controls for each case. For example, for a case who commenced ART in June 2005, we used an electronic ART database to find two controls who were also initiated on ART in June 2005 with similar gender and within ±5 years of age of the case.

### Variables and measures

Sociodemographic and clinical characteristics of participants were assessed for their possible association with the outcome variable (ART failure) (Table 1). The variables measured before ART initiation were baseline CD4 lymphocyte count and WHO HIV stage, whereas all the other variables were retrospectively measured during patient follow-up after ART was started.

**Table 1:** Socio-demographic and baseline clinical characteristic of adult HIV-positive patients, Southeast Ethiopia, 2015–2017

Variables	Total (*N* = 273)	Patients with ART failure (Cases) (*N* = 91)	Patients without ART failure (Controls) (*N* = 182)
Gender-male	149 (55%)	52 (19%)	97 (36%)
Median age in years (IQR)	40 (11)	39 (10)	40 (12)
Religion			
Orthodox Christian	187 (69%)	62 (23%)	125 (46%)
Muslim	160 (21%)	20 (7%)	37 (14%)
Protestant	27 (10%)	9 (3%)	18 (7%)
Others	2 (1%)	0 (0%)	2 (1%)
Marital status			
Married	160 (59%)	47 (17%)	113 (41%)
Not married	113 (41%)	44 (16%)	69 (25%)
Family size			
≤ 5 family members	217 (80%)	76 (28%)	141 (52%)
> 5 family members	54 (20%)	13 (5%)	41 (15%)
Educational status			
Illiterate	39 (14%)	12 (4%)	27 (10%)
Literate	234 (86%)	79 (29%)	155 (57%)
Employment			
Employed	267 (98%)	87 (32%)	180 (66%)
Unemployed	6 (2%)	4 (1%)	2 (1%)
Residence area			
Rural	67 (25%)	28 (10%)	39 (14%)
Urban	206 (76%)	63 (23%)	143 (52%)
Khat** chewing			
Yes	10 (4%)	4 (4%)	6 (3%)
No	263 (96%)	87 (96%)	176 (97%)
Alcohol use			
Yes	7 (3%)	1 (1%)	6 (3%)
No	266 (97%)	90 (99%)	176 (97%)
Smoke cigarettes			
Yes	6 (2%)	3 (3%)	3 (2%)
No	267 (98%)	88 (97%)	179 (98%)
Main diet			
Wheat or barley	40 (15%)	18 (20%)	22 (12%)
Teff	165 (60%)	54 (60%)	111 (61%)
Others	68 (25%)	19 (20%)	49 (27%)
Poor drug absorption			
Main diet wheat or barley with diarrhea	3 (1%)	3 (3%)	0 (0%)
Main diet wheat or barley without diarrhea	37 (14%)	15 (16%)	22 (12%)
Duration on ART median in months (IQR)	69 (41)	64 (39)	72 (36)
Baseline CD4 lymphocyte count			
< 50 cells/mm^3^	33 (12%)	20 (22%)	13 (7%)
≥ 50 cells/mm^3^	240 (88%)	71 (78%)	169 (93%)
Baseline WHO HIV stage			
Stage 1	26 (10%)	8 (9%)	18 (10%)
Stage 2	93 (34%)	22 (24%)	71 (39%)
Stage 3	140 (51%)	52 (57%)	88 (48%)
Stage 4	14 (5%)	9 (10%)	5 (3%)
ART regimen			
AZT based	130 (48%)	57 (63%)	96 (53%)
TDF based	143 (52%)	34 (37%)	86 (47%)
TB treatment while taking ART			
Never	231 (85%)	58 (64%)	173 (95%)
Once	33 (12%)	26 (28%)	7 (4%)
Two times	8 (3%)	6 (7%)	2 (1%)
Three or more	1 (0%)	1 (1%)	0 (0%)
Never discontinued ART	212 (78%)	49 (54%)	163 (90%)
Discontinued ART (by duration)	61 (22%)	42 (46%)	19 (10%)
< 1 month	32 (12%)	23 (25%)	9 (5%)
> 1 month	29 (10%)	19 (21%)	10 (5%)
Missed ART follow-up			
Never	233 (85%)	70 (77%)	163 (89.5%)
Sometimes	32 (12%)	14 (15%)	18 (10%)
Often	8 (3%)	7 (8%)	1 (0.5%)
Repeated or persistent diarrhea			
Yes	24 (9%)	16 (18%)	8 (4%)
No	249 (91%)	75 (82%)	174 (96%)
Psychiatric illness			
Yes	10 (4%)	6 (7%)	4 (2%)
No	263 (96%)	85 (93%)	178 (98%)
Dyspepsia			
Yes	82 (30%)	31 (34%)	51 (28%)
No	191 (70%)	60 (66%)	131 (72%)
Diabetic			
Yes	1 (0.4%)	1 (1%)	0 (0%)
No	272 (99.6%)	90 (99%)	182 (100%)
Hypertensive			
Yes	3 (1%)	0 (0%)	3 (2%)
No	270 (99%)	91 (100%)	179 (98%)
Stool *H. pylori* antigen positive			
Yes	64 (23%)	18 (20%)	46 (25%)
No	209 (77%)	73 (80%)	136 (75%)
HBsAg positive			
Yes	12 (4%)	3 (3%)	9 (5%)
No	261 (96%)	88 (97%)	173 (95%)
Anti-HCV positive			
Yes	5 (2%)	1 (1%)	4 (2%)
No	268 (98%)	90 (99%)	174 (96%)
VRDL positive			
Yes	56 (20%)	13 (14%)	43 (24%)
No	217 (80%)	78 (86%)	139 (76%)

Note: *ART*: Antiretroviral Therapy; *AZT*: Azidothymidine (Zidovudine); *HCV*: Hepatitis C virus; H. pylori: Helicobacter pylori; *HBsAg*: Hepatitis B surface antigen; *IQR*: interquartile range; *TDF*: Tenofovir; *TB*: tuberculosis; *VDRL*: Venereal disease research laboratory; *WHO*: World Health Organization. *Teff (*Eragrostis tef*) is a gluten-free cereal traditionally grown in Ethiopia [25]. **Khat *(Catha edulis)* is a plant native to the Horn of Africa and the Arabian Peninsula which contains psychoactive substances that have a high abuse potential [26]

Duration on ART was defined as the total time (in months) a patient was on ART since initial enrolment into ART care excluding the period of interruptions. Discontinuation of ART for a patient currently on ART was defined as having a history of complete stoppage of ART medications for any duration; whereas missed ART follow-up meant that a patient missed a drug refill date. The ART regimen for cases was their first-line ART regimen, not their current second-line regimen.

Repeated or persistent diarrhea was defined as ≥2 episodes of diarrhea or a single episode lasting more than 2 weeks, respectively. We used the term psychiatric illness in patients who were diagnosed with a disorder listed in the Diagnostic and Statistical Manual of Mental Disorders IV [24]. Tuberculosis (TB) co-infection was defined as an active TB disease requiring a curative therapy with anti-TB medications. Alcohol use was defined as the use of alcoholic beverages in any amount. A main-diet constitutes the usual sum of food consumed by participants in a stable manner for years. Poor drug absorption was assumed to be present in those patients who experienced diarrhea (repeated or persistent) and frequently eat wheat or barely. For variables measuring co-existing chronic infections, we presumed that these diseases were either acquired during a similar high-risk behavior that led to the acquisition of HIV itself (hepatitis B virus (HBV), hepatitis C virus (HCV), and syphilis) or acquired early in life (HBV and Helicobacter pylori (*H. pylori*) infection).

### Data collection tool and procedure

The data collection tools were interview-questionnaire, electronic ART database, patient chart review and virologic test for selection of controls. In addition, other laboratory tests were also performed to detect chronic carrier state for infections, including HBV, HCV, *H. pylori* and Venereal disease research laboratory (VDRL) tests for both cases and controls.

The questionnaire was first developed in English and translated into local languages, Oromiffa and Amharic, and cross-checked for consistency. It was piloted, modified and validated. Training was provided for the study team before the start of the project. The research team included the three investigators, two registered nurses who assisted patients to complete the questionnaire, five laboratory technicians and an IT professional to record all the observations into Epi-info software daily. The investigators continuously monitored the data collection process throughout the study. Data completeness was regularly checked, and any missing data were obtained on the subsequent follow-up of patients.

### Data processing and analysis

Data from Epi-Info was exported to SPSS statistics version 23.0. Descriptive statistics were used to present socio-demographic and clinical characteristics. Bivariate logistic regression analysis was carried out, and independent variables with *p*-values of ≤0.2 were included in multivariate logistic regression analysis. Prior to the multivariate analysis, multicollinearity diagnostics was performed, and there were no significant interactions between independent variables. Adjusted odds ratios (AOR) with 95% confidence intervals (CIs) were used as an effect measure. A *p*-value of equal or less than 0.05 was considered as significant. The matching factors (age, sex and duration of ART) were included in the adjusted analysis.

### Ethics approval

Ethical approval was obtained from the Arsi University College of Health Sciences Ethics Review Board. Informed consent was obtained from study participants. Information gathered from respondents was treated confidentially, and the norm of the community was considered and respected in the process of data collection. Patients with abnormal findings were referred to the treating physician and were provided with the appropriate care.

## Results

### Socio-demographic characteristics of participants

Of all 273 study participants, 55% were males. The median age of the participants was 40 years. The median duration of treatment was 69 months. Most of the participants were Orthodox Christian 187 (69%), married 160 (59%), and literate 234 (86%). Approximately three-quarters of patients were living in the urban area, and 267 (98%) were employed (Table 1).

When we compare the characteristics of cases with controls in terms of age, sex and duration of ART; they were roughly comparable in a 1:2 ratio. Compared with controls, cases were six times more affected with active tuberculosis and four times more likely to have persistent diarrhea (Tables 1 and 2).

**Table 2:** Risk factors for ART failure in adult HIV-positive patients, Southeast Ethiopia, 2015–2017

Variables	Bivariate analysis			Multivariate analysis		
	COR	95% CI	P-value	AOR	95% CI	P-value
Illiterate	0.9	0.4–1.8	0.714			
Small family ≤5	1.5	0.8–2.8	0.245			
Residence in rural area	1.6	0.9–2.9	0.092	1.4	0.6–2.9	0.396
Unemployed	4.1	0.7–23	0.105	1.5	0.1–27.2	0.775
Unmarried	1.5	0.9–2.6	0.100	1.4	0.7–2.7	0.379
Alcohol	3.1	0.4–25.9	0.303			
smoking cigarette	2.0	0.4–10.3	0.390			
Khat** chewing	1.3	0.4–4.9	0.650			
Main diet wheat or barley vs other food	1.8	0.9–3.5	0.093	2.3	0.9–5.4	0.064
Main diet teff vs wheat or barley	0.6	0.3–1.2	0.147			
Baseline CD4 lymphocyte count ≤50 cells/mm^3^	3.7	1.7–7.8	< 0.001	3.8	1.5–9.6	0.005
Baseline WHO stage 4	3.9	1.3–12.0	0.018	0.3	0.1–1.4	0.138
Discontinuation of ART	7.4	3.9–13.8	< 0.001	9.8	4.0–23.8	< 0.001
< 1 month	8.5	3.7–19.6	< 0.001			
> 1 month	6.3	2.8–14.5	< 0.001			
AZT based regimen	1.5	0.9–2.5	0.121	1.3	0.7–2.6	0.392
Missed ART follow-up	2.6	1.3–5.1	0.007	1.6	0.6–4.8	0.362
Repeated or persistent diarrhea	4.6	1.9–11.3	< 0.001	4.4	1.5–13.2	0.007
Dyspepsia	1.3	0.8–2.3	0.305			
Psychiatric illness	3.1	0.9–11.4	0.082	0.6	0.3–1.5	0.272
Anti-HCV positive	2.0	0.2–18.4	0.531			
HBsAg positive	1.5	0.04–5.8	0.534			
H. pylori antigen positive	0.7	0.4–1.3	0.314			
VDRL positive	0.5	0.3_1.1	0.074	0.6	0.3–1.5	0.272

Note: *ART*: Antiretroviral Therapy; *AZT*: Azidothymidine (Zidovudine); *CI*: Confidence interval; *COR*: Crude odds ratio; *HCV*: Hepatitis C virus; *H*. pylori: Helicobacter pylori; *HBsAg*: Hepatitis B surface antigen; *VDRL*: Venereal disease research laboratory; *WHO*: World Health Organization. *Teff (*Eragrostis tef*) is a gluten-free cereal traditionally grown in Ethiopia [25]. ** Khat *(Catha edulis)* is a plant native to the Horn of Africa and the Arabian Peninsula and contains psychoactive substances that have a high abuse potential [26]

## Discussion

This study identified discontinuation of ART, baseline CD4 lymphocyte count ≤50 cells/mm^3^ and persistent diarrhea as independent factors for first-line ART failure. The risk of ART failure was high and comparable whether the duration of ART discontinuation was greater or less than 1 month. Frequent eating of a diet made of wheat or barely showed a trend of statistically significant association with first-line ART failure.

Patients who did experience first-line ART failure were nearly 10 times more likely to have a history of total ART discontinuation (AOR = 9.8, 95% CI: 4.0–23.8) than those who did not. The risk of ART failure in patients who discontinued ART for more than 1 month (COR = 6.3, 95% CI: 2.8–14.5) was comparable with those who discontinued it for less than 1 month (COR = 8.5, 95% CI: 3.7–19.6). The association between ART failure and drug discontinuation was also significant in other studies conducted in a similar setting. Comparable findings were reported by case-control studies from Zimbabwe (AOR = 5.1, 95% CI 2.8–10.0) [19], Tanzania (AOR = 12.0, 95% CI 2.1–69.3) [27] and the northern part of Ethiopia (AOR = 15.80, 95% CI: 6.9–36.5) [28].

Patients who were initiated on ART at an advanced stage of HIV/AIDS (baseline CD4 lymphocyte count ≤50 cells/mm^3)^ were four times more likely to experience first-line ART failure (AOR = 3.8, 95% CI: 1.5–9.6) in this study. Several studies reported that starting ART late in patients with advanced immunosuppression is associated with antiretroviral treatment failure [17, 29–33].

Persistent diarrhea was also found to be an independent predictor of ART failure (AOR = 4.4, 95% CI: 1.5–13.2) as documented in other studies [34, 35]. Poor absorption of ART medications associated with a general malabsorption state could occur in patients with persistent diarrhea. However, this finding could be an overestimation as persistent diarrhea could be a manifestation of ART failure itself with disproportionately more diarrheal cases among the cases group.

Furthermore, frequent eating of wheat or barley-based meal (AOR = 2.3, 95% CI: 0.9–5.4) showed a trend to be a risk factor for first-line ART failure (*p*-value < 0.064) compared with other food. It is important to note that wheat and barley are gluten-containing foods, and both foods accelerate gastrointestinal transit [36, 37]. Compared to barley and wheat, frequent eating of teff, which is a gluten-free staple grain of Ethiopian cuisine [25], showed a decreased odds ratio for ART failure (COR = 0.6, 95% CI: 0.3–1.2). To our knowledge, there are no previous studies that documented frequent eating of wheat and barley-based diet as a risk factor for first-line ART failure, and further studies with larger sample size are required before generalizing.

Our study has several limitations. Most of the independent variables were self-reported. Therefore, memory bias and imperfect temporal precedence could have under or overestimated our results. The smaller study population and sensitivity and specificity of laboratory tests might also have affected the accuracy of our findings.

## Conclusions

Discontinuation of ART drugs, persistent diarrhea and baseline CD4 lymphocyte count ≤50 cells/mm^3^ were strongly associated with first-line ART failure. Our findings underscore the importance of avoiding ART discontinuation of any duration to prevent treatment failure. Wheat or barley as the main diet could be a possible cause for ART failure; however, further studies are needed.

## Data Availability

The datasets generated and/or analyzed during the current study are not publicly available due to the sensitivity of the topic and hence to ensure confidentiality of the information but are available from the corresponding author on reasonable request.

## Abbreviations

AOR: Adjusted odds ratio
ART: Antiretroviral Therapy
AZT: Azidothymidine (Zidovudine)
CI: Confidence interval
COR: Crude odds ratio
*H. pylori*: Helicobacter pylori
HBsAg: Hepatitis B virus surface antigen
HBV: Hepatitis B virus
HCV: Hepatitis C virus
HIV: Human Immunodeficiency Virus
mL: milliliter
NNRTI: Non-nucleoside reverse transcriptase inhibitor
SD: Standard deviation
TB: Tuberculosis
VDRL: Venereal disease research laboratory
WHO: World Health Organization
